# Identification of a novel DNA aptamer that selectively targets lung cancer serum†

**DOI:** 10.1039/d1ra06233f

**Published:** 2021-10-15

**Authors:** Yunwang Zhao, Lei He, Baihai Huang, Weidong Zhang, Ailing Hu, Baolin Li, Shiqi Liao, Na Wang

**Affiliations:** The First Hospital of Qinhuangdao Qinhuangdao 066000 China zhaoyunwang1234@163.com wangncqhd@163.com +86-0335-590-8439; Guangdong Provincial Key Laboratory of Synthetic Genomics, Shenzhen Institute of Advanced Technology, Chinese Academy of Sciences Shenzhen 518055 China; Key Laboratory of Molecular and Cellular Systems Biology, College of Life Sciences, Tianjin Normal University Tianjin 300387 China; College of Life Sciences, Lanzhou University Lanzhou 730000 China

## Abstract

Lung cancer is the leading cause of cancer-related deaths worldwide. Early diagnosis and treatment is critical to improving the 5 year survival rate of lung cancer. The identification of new options for early-stage diagnosis and therapy of lung cancer still represents a crucial challenge. Therefore, a new diagnostic method is urgently needed. In this study, we used a new modified SELEX, called serum-SELEX, to isolate aptamers that can specifically bind lung cancer serum, without any prior knowledge of their target. Among the obtained candidate aptamer sequences, Ap-LC-19 was identified as the optimal aptamer probe with the lowest dissociation constant (*K*_d_) value of 15 ± 8.6 nM and higher affinity assessed by qPCR. Furthermore, this molecule could be a suitable aptamer for lung cancer serum and could be used as a recognition element in aptamer-based biosensors for efficient early diagnosis of lung cancer or as an innovative tool for targeted therapy. In addition, we performed MALDI-TOF MS followed by secondary peptide sequencing MS analysis for the identification of the aptamer targeted proteins. CLEC3B could be useful biomarkers for early detection of lung cancer and in monitoring its evolution.

## Abstract 2

Lung cancer is the leading cause of cancer-related deaths worldwide.

## Graphical abstract

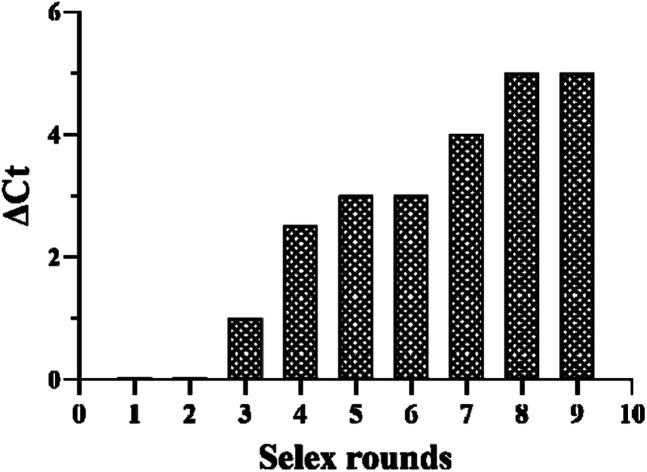

## Introduction

Lung cancer is the leading cause of cancer-related deaths worldwide.^1^ Lung cancer patients have a poor prognosis and a 5 year survival rate of less than 20%.^2^ However, patients diagnosed at an early stage and who have surgery experience an 86% overall 5 year survival. Early diagnosis and therapy is critical to improving the survival rate of lung cancer.^3^ Several conventional methods have been developed for the detection of lung cancer. Most of these methods are high cost,^4^ time consuming^5^ and low sensitivity,^6^ but also require sophisticated instruments. To overcome these factors, new methods and reagents should be developed for lung cancer diagnosis.

A promising class of targeted molecules is represented by aptamers. Aptamers are short single-stranded nucleic acids (ssDNA or RNA) molecules which can bind target molecules with high affinity and specificity, and are generated by the SELEX technology (systematic evolution of ligands by exponential enrichment).^7,8^ Aptamers possess many obvious features superior to antibody including high specificity, high stability, low toxicity,^9^ cost-effectiveness,^10^ easy synthesis^11^ and modification,^12^ and no immunogenicity.^13^

Serum is among the most available unpurified biological mixtures, which contain proteins nearly from all organs,^14^ tissues and cells that play critical roles in exploration of the occurrence,^15^ development, diagnosis and therapy of diseases.^16,17^ Theoretically, the use of serum as a compound target may allow selecting a large group of aptamers to known and unknown disease-related biomarkers. Li *et al.* used the acetonitrile precipitation method to remove high-abundance proteins in colorectal cancer serum as the screening target, and obtained its specific aptamers after 10 rounds of SELEX screening.^18^ As such, the serum aptamer screening method is effective for developing aptamer-based biosensors selection protocol.

In the present study, we explored the use of aptamers to specifically target lung cancer serum, discriminating against them from normal serum and other tumor serum. We used a new modified SELEX (serum-SELEX) to isolate aptamer that can specifically bind lung cancer serum. Four aptamer sequences, Ap-LC-2, Ap-LC-3, Ap-LC-6 and Ap-LC-19, were identified to bind to target serum with *K*_d_ value of 22 ± 7.2 nM, 29 ± 8.9 nM, 35 ± 6.3 nM and 15 ± 8.6 nM, respectively. Among the four aptamers, Ap-LC-19 was identified as the optimal aptamer probe with the lowest dissociation constant *K*_d_ value of 15 ± 8.6 nM and higher specificity assessed by qPCR. In summary, our results show that these four aptamers had potential application for lung cancer early diagnosis and targeted therapy. We also performed MALDI-TOF MS followed by secondary peptide sequencing MS analysis for the identification of the aptamer targeted proteins. CLEC3B could be useful biomarkers for early detection of lung cancer and in monitoring its evolution.

## Materials and methods

### Random DNA library, primers, reagents and serum

A ssDNA 88 nt randomized oligonucleotide library (ssDNA library sequence: 5′-CTATAGCAATGGTACGGTACTTCC-N40-CAAAAGTGCACGCTACTTTGCTAA-3′) was used as an initial library. The sequences of oligonucleotides consist of a central region of 40 random oligonucleotides anked by two fixed primer regions at both ends for qPCR amplification. Forward primer, 5′-CTATAGCAATGGTACGGTACTTCC-3′ and reverse primer, 5′-biotin-TTAGCAAAGTAGCGTGCACTTTTG-3. Both the library and the primers were chemically synthesized by Sangon Biotech (Shanghai, China). Carboxylated magnetic beads (MBs) were obtained from Thermo Fisher Scientific (USA).

The lung cancer serum (collecting 100 cases of early lung cancer with pathological diagnosis), normal serum, gastric cancer serum, colorectal cancer serum and hepatocellular carcinoma serum were supplied by the first hospital of Qinhuangdao, Hebei Province in China. The study was approved by the Ethics Committee of the First Hospital of Qinhuangdao (2020B018). The study participants provided written informed consent. The reporting of this study confirms with STROBE guidelines.^19^

### Serum-SELEX approach

Leftover serum specimens (initially drawn for routine laboratory tests) and clinical data before therapy were collected from patients with lung cancer hospitalized in the first hospital of Qinhuangdao and from normal controls (NC) who came for health examination at the hospital (Table S1†).

To overcome tumor heterogeneity and ensure the diversity of specific targets of lung cancer serum for SELEX, fifty confirmed lung cancer cases were randomly selected and were mixed to prepare pooled lung cancer serum, and the volume of pooled serum was prepared according to the need of experiments. Pooled normal serum was also prepared similarly.

To screen aptamers against lung cancer serum. 1 mL of the MBs (5 × 10^9^ in 1 mL) was dispersed in 1 mL of pooled lung cancer serum into a 10 mL EP tube and then incubated at 37 °C for 2 h with gentle rotation. After incubation, a magnet was used to immobilize the pooled lung cancer serum-MBs complexes and then we removed the unbound serum supernatant. The pooled lung cancer serum-MBs complexes were washed three times with 1 mL of binding buffer (BB, 50 mM Tris–HCl, 5 mM 114 KCl, 100 mM NaCl, 1 mM MgCl_2_, pH 7.4) and the complexes were subsequently collected by magnetic separation. Followed by adding 2 mL blocking solution (To 1 L of BB, and 100 mg tRNA, 1 g BSA) to the MBs to block their surface sites.^20,21^ We finally obtained MBs covered with the pooled lung cancer serum. We used pooled normal serum as a counter-SELEX target, so we used the same procedure to immobilize pooled normal serum on the MBs. Before each cycle of SELEX, briefly, the initial ssDNA library (1 OD, 2.5 nmol) was dissolved in 3 mL BB solution and subjected to denaturation steps of 95 °C for 5 min, and then kept on ice for 10 min, to form a unique 3D structure. The ssDNA library was then incubated for 1 h at 37 °C with pooled normal serum-MBs complexes with gentle rotation (counter-selection step). After pooled normal serum incubation with evolved ssDNA library, unbound ssDNA was recovered by using a magnetic separator and incubated with pooled lung cancer serum-MBs complexes for 30 min with gentle rotation (selection step) and then separated by magnetic separator to remove unbound aptamers, followed by washing three times (thrice) with 1 mL of BB. Afterward, in the partition step, the selection step MBs were resuspended in 200 μL ddH_2_O and heated for 10 min at 95 °C to release aptamers bound to the pooled lung cancer serum. After isolating the MBs by magnets, the ssDNA supernatant was collected and used as the sublibrary for next-round selection. Subsequently, the recovered pooled lung cancer serum-binding ssDNA aptamers were amplified by qPCR using forward primer and biotin-labeled reverse primer under the following conditions: 95 °C for 2 min, 60 °C for 34 s and 72 °C for 1 min with 20 or 25 cycles. The PCR products were separated by streptavidin-coated MBs (500 μL), then it was mixed with formamide solution (5 wt%, 1 mL)^22^ and incubated for 10 min at 40 °C. After discarding the supernatant, the streptavidin-coated MBs were washed twice with BB, followed by adding 200 μL ddH_2_O, and then heated at 95 °C for 10 min to separate DNA and streptavidin-coated MBs. The supernatant was collected and used as the sublibrary later for the next round selection. During selection, the amount of the eluted target serum ssDNA pools to target serum was monitored the screening process according to the Δ*C*_t_ (the difference value between the *C*_t_ value of the collected ssDNA binding to the serum from lung cancer patients and the collected ssDNA binding to the serum from the healthy people) value through qPCR.

### Sequence analysis

The enriched aptamer sequences were sent to Sangon Biotechnology (Shanghai, China) for high-throughput sequencing by an Illumina high-throughput sequencing platform. The results of aptamers sequencing were analyzed by DNAMAN software. The most frequent and enriched aptamer sequences were chosen for further analysis. Finally, according to the algorithm of DNA minimum free energy, the secondary structures of aptamers were analyzed using NUPACK.^23,24^

### Dissociation constant (*K*_d_) measurement

A certain amount of MBs (1.2 mL) (the volume ration between MBs and pooled lung cancer serum was 1 : 1) were added and incubated at 37 °C for 1 h as positive control. After magnetic separation, unbound serum was removed by using a magnetic separator, followed by washing three times with 200 μL BB. MBs coated by pooled normal serum were used as a negative control. The pooled lung cancer serum-MBs complexes were equally divided into six parts in six 1.5 mL EP tubes. These complexes were incubated with different concentrations (0, 10, 25, 50, 100, 250 and 500 nM as final concentrations) of fluorescein-labeled (5-FAM) ssDNA in 200 μL of BB for 1 h at 37 °C with gentle rotation, respectively. The MBs-serum-binding aptamers complexes were collected by using a magnetic separator. After washing five times with the BB, the serum-MBs-aptamers complexes were resuspended in 1 mL of BB. Finally, the fluorescence intensity of each sample was measured by a fluorospectrophotometer.^25–27^ To estimate the dissociation constant *K*_d_ values of the selected ssDNA sequences to lung cancer serum, the fluorescence intensity *versus* the added ssDNA concentration was plotted. These data showing the binding saturation curves were fitted by nonlinear regression analysis. The *K*_d_ values were analyzed with Sigma Plot 12.5 software using the following equation. *Y* = *B*_max_*X*/(*K*_d_ + *X*).^28,29^ Where *Y* represents the average value of fluorescence due to binding with FAM-labeled aptamers. *X* is considered as the aptamer concentration and *B*_max_ is the maximum binding capacity of a specific aptamer. All assays were performed in triplicate.

### Circular dichroism (CD) measurement

Four candidate aptamers (Ap-LC-2, Ap-LC-3, Ap-LC-6 and Ap-LC-19) were resuspended in 1 μM with 20 mM Na_3_PO_4_ (pH 7.4, contained 50 mM KCl or NaCl), and incubated with 10 μM lung cancer serum for 30 min at 37 °C, and then heated at 95 °C for 5 min, followed by fast cooling on ice. Normal serum was used as a negative control. CD measurements were carried out on a JASCO J-810 Spectrometer (JASCO, Tokyo, Japan) in a quartz cell with an optical a 1 cm path length cuvette. The CD spectra was obtained by taking the average of two scans made from 220–320 nm.

### Candidate sequence detection in clinical samples

The diagnostic performances of four aptamers, Ap-LC-2, Ap-LC-3, Ap-LC-6 and Ap-LC-19, were further evaluated with an enlarged serum sample. 200 μL of lung cancer serum were immobilized onto 100 μL of MBs *via* 2 h at 37 °C for 2 h with gentle rotation incubation for aptamer identification. As a negative selection control, an equal amount of normal serum was immobilized on MBs *via* an identical method as lung cancer serum. For binding assays on each lung cancer serum aptamer candidate in BB was heated to 95 °C for 10 min then cooled to 0 °C for 10 min. An equal amount of the aptamer solution was then incubated with lung cancer serum-MBs complexes and normal serum-MBs complexes respectively for 30 minutes with gentle rotation at 37 °C. Unbound ssDNA was removed with the magnetic separator by washing four times with BB. Lung cancer serum-bound aptamers and normal serum-bound aptamers were denatured and eluted with ddH_2_O at 95 °C, respectively. Recovered aptamers were amplified by qPCR. The collected fifty cases of lung cancer sera and fifty cases of normal sera were specifically identified according to the above steps. All assays were performed in triplicate.

### Specificity test

The specificity was determined using qPCR test. The serum samples included 10 patients with gastric cancer, 10 patients with lung cancer, 10 patients with colorectal cancer, 10 patients with hepatocellular carcinoma, and 10 healthy or control patients. 100 μL of MBs and 200 μL of different serum were mixed and incubated at 37 °C for 1 h, respectively. A negative control normal serum was performed simultaneously. Following magnetic separation, different serum-MBs were washed three times with BB for 1 min each time, 150 nM of treated candidate and different serum-MBs complexes were mixed and incubated at 37 °C for 30 min, respectively. The supernatant solution was discarded, the different serum MBs-aptamers complexes were washed three times with the BB for 1 min each time. Followed by adding 200 μL ddH_2_O, and then heated at 95 °C for 10 min to separate DNA and different serum-MBs complexes. The supernatant was collected and amplified with qPCR. The qPCR operating system is as follows: 95 °C for 2 min, 60 °C for 34 s and 72 °C for 1 min with 40 cycles.

### Apt-LC-19 probe for identification of target protein

The sample preparation and MALDI-TOF MS analytical procedures were the same as reported by Zheng *et al.*^22^ Briely, the Apt-LC-19 aptamer (5 × 10^−6^ mol L^−1^, 20 μL) and the pooled lung cancer serum (20 μL) were incubated respectively for 30 min at 37 °C, the pooled normal serum as a negative control. The initial ssDNA library binds to pooled lung cancer serum and pooled normal serum as randomized sequence. Then, these complexes were separated on 8% native polyacrylamide gel. The gel retarded band was excised and the protein components in it were recovered, digested with trypsin digest solution (proteomics grade, Roche). The resulting digests were analyzed by 4800 Plus MALDI-TOF MS (ABSciex, Applied Biosystems, Foster City, CA) and then analyzed by MS with secondary peptide sequencing. The identification of the mixture proteins was searched in S. Mansoni Swiss-Prot protein database. The corresponding position in the control lane (the complex of the initial ssDNA library and pooled lung cancer serum, the complex of the Apt-LC-19 and pooled normal serum) was excised and identified by MS simultaneously to exclude the protein mobilized similarly with the complex. The candidate serological biomarkers of lung cancer were thus gained.

## Results and discussion

### *In vitro* aptamer selection using serum-SELEX

**Scheme 1:**
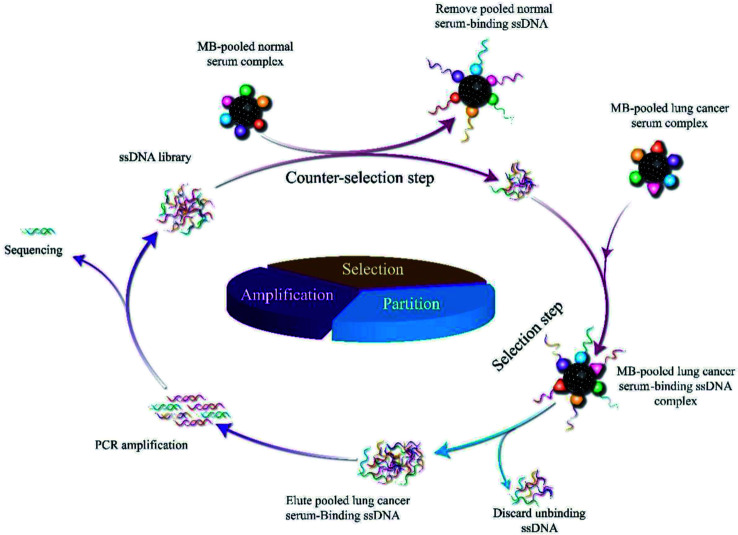
Schematic illustration of the serum-SELEX process.

The serum-SELEX steps are schematically shown in Scheme 1. In order to select aptamers able to specifically recognize the lung cancer serum, we developed a new serum-SELEX for the first time. Specifically, in each round, small fragments of blocking solution were incubated with MBs-coupled pooled lung cancer serum to block the non-specific binding sites. We performed nine rounds of SELEX. A total of nine rounds of counter-selection step and selection step were performed until the ssDNAs binding to targeting pooled lung cancer serum was achieved. During SELEX, the counter-selection step was introduced to remove nonspecific or weak binding sequences and was examined to enhance the selectivity of aptamers to the target serum using pooled normal serum. Each round of selection pressure was gradually enhanced (Table 1). During the selection process, in order to select aptamers with high specificity and high affinity, the input sublibrary decreased from 2500 pmol to 1007.3 pmol and the ratios of sublibrary to serum were gradually increased as the number of selection rounds increased. Progressively increasing the selection pressure by changing incubation time (from 60 min to 30 min) and washing conditions.

**Table 1:** The pooled lung cancer serum aptamers SELEX screening conditions

Rounds	Amount of MBs (mL)	ssDNA library (pmol)	ssDNA library into each round (pmol)	Lung cancer mixed serum volume (mL)	Ratio of MBs-serum to library	Incubation time (min)
1	1	2500	2500	1	2	60
2	1	1665.5	1665.5	1	2	60
3	1	2870.6	1870.6	1	4	50
4	1	2147.6	1798.7	1	4	50
5	1	1543.3	1438.9	0.8	6	50
6	1	1856.3	1618.9	0.8	6	40
7	1	1766.4	1439.0	0.6	8	40
8	1	1082.9	1079.3	0.6	8	40
9	1	1010.9	1007.3	0.6	10	30

**Fig. 1:**
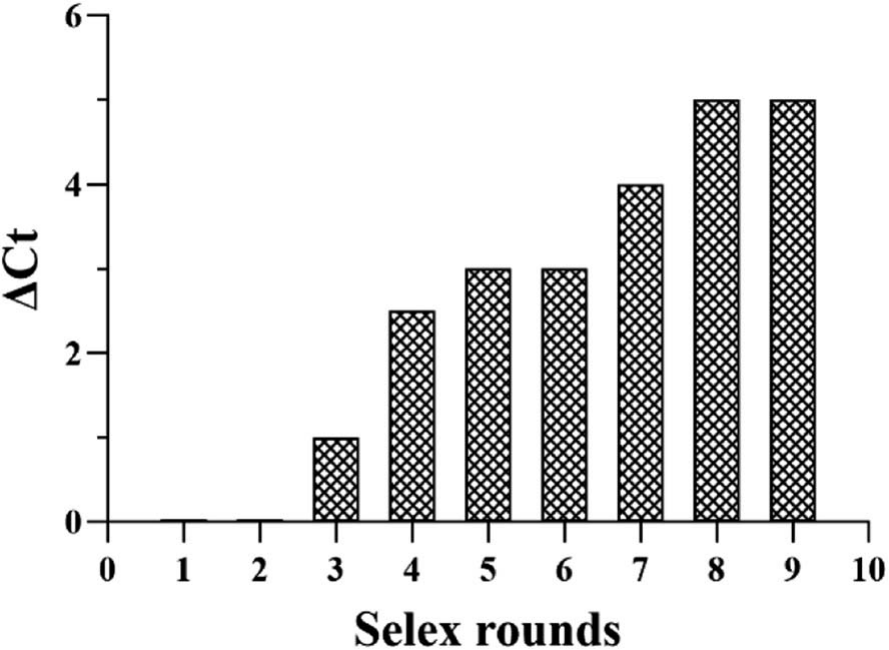
The binding affinity of the aptamer pools from the selected rounds were calculated using the qPCR method.

To further evaluate our protocol, qPCR assays were used to monitor the selection process according to the Δ*C*_t_ values. The greater is the Δ*C*_t_ value, the higher is the enrichment of the candidate aptamers with high affinity to the target serum. The SELEX process reached a plateau at 8th round. As shown in Fig. 1, according to binding assay, sequences with high binding affinities for the pooled lung cancer serum were gradually enriched, and no significant improvement in Δ*C*_t_ was observed when the number of selection rounds was greater than 8th round. The above results confirm that the ssDNA library of the 8th round had been enriched. The saturation of the binding sites on the pooled lung cancer serum-coated MBs might have contributed to the constant Δ*C*_t_ values after eight selection rounds. Therefore, the result suggests that with respect to initial ssDNA library, pool 9 was enriched for aptamers against pooled lung cancer serum. The serum-SELEX was then stopped at the 9th round.

### High-throughput sequencing and analysis

In order to isolate more comprehensive and true information about individual aptamers that may specifically recognize pooled lung cancer serum, the terminal DNA library (ssDNA pool 9) from serum-SELEX was amplified and then used for high-throughput sequencing (HTS).

After sequencing, ten potential pooled lung cancer serum aptamers were screened and respectively named as Ap-LC-2, Ap-LC-3, Ap-LC-6, Ap-LC-19, Ap-LC-25, Ap-LC-32, Ap-LC-51, Ap-LC-54, Ap-LC-69 and Ap-LC-76 for the SELEX. The specific sequences of the candidate aptamers are shown in Table 2.

**Table 2:** The pooled lung cancer serum aptamer sequences

Aptamer	Nucleic acid sequence (5′–3′)	Abundance (%)	*K*_d_ (nmol L^−1^)
Ap-LC-2	5′-C̲T̲A̲T̲A̲G̲C̲A̲A̲T̲G̲G̲T̲A̲C̲G̲G̲T̲A̲C̲T̲T̲C̲C̲CTTTGGTACGGATCTTCCAAGCTAACCCTACTCT	12	22 ± 7.2
	GCGCGCC̲A̲A̲A̲A̲G̲T̲G̲C̲A̲C̲G̲C̲T̲A̲C̲T̲T̲T̲G̲C̲T̲A̲A̲-3′		
Ap-LC-3	5′-C̲T̲A̲T̲A̲G̲C̲A̲A̲T̲G̲G̲T̲A̲C̲G̲G̲T̲A̲C̲T̲T̲C̲C̲CCACGGCACGTTCACTGTAGCGCACGCTGGACA	17	29 ± 8.9
	TCCCACAC̲A̲A̲A̲A̲G̲T̲G̲C̲A̲C̲G̲C̲T̲A̲C̲T̲T̲T̲G̲C̲T̲A̲A̲-3′		
Ap-LC-6	5′-C̲T̲A̲T̲A̲G̲C̲A̲A̲T̲G̲G̲T̲A̲C̲G̲G̲T̲A̲C̲T̲T̲C̲C̲CCAAGCAATCCCGGATCTGCGCGCACCTCAGAT	13	35 ± 6.3
	GCGCTGCC̲A̲A̲A̲A̲G̲T̲G̲C̲A̲C̲G̲C̲T̲A̲C̲T̲T̲T̲G̲C̲T̲A̲A̲-3′		
Ap-LC-19	5′-C̲T̲A̲T̲A̲G̲C̲A̲A̲T̲G̲G̲T̲A̲C̲G̲G̲T̲A̲C̲T̲T̲C̲C̲ATGGTAGGCTACAACCAAGCTAAGGGCATCTGC	26	15 ± 8.6
	GCGCTCC̲A̲A̲A̲A̲G̲T̲G̲C̲A̲C̲G̲C̲T̲A̲C̲T̲T̲T̲G̲C̲T̲A̲A̲-3′		
Ap-LC-25	5′-C̲T̲A̲T̲A̲G̲C̲A̲A̲T̲G̲G̲T̲A̲C̲G̲G̲T̲A̲C̲T̲T̲C̲C̲AAGCTAACCCACTTCCGCGCGCTGGCCGAACAG	3	76 ± 8.4
	TTAGCGCC̲A̲A̲A̲A̲G̲T̲G̲C̲A̲C̲G̲C̲T̲A̲C̲T̲T̲T̲G̲C̲T̲A̲A̲-3′		
Ap-LC-32	5′-C̲T̲A̲T̲A̲G̲C̲A̲A̲T̲G̲G̲T̲A̲C̲G̲G̲T̲A̲C̲T̲T̲C̲C̲TACGACTCACATATAGGGCTAAGGGACATCTCG	2	106 ± 7.2
	GCGCAGCC̲A̲A̲A̲A̲G̲T̲G̲C̲A̲C̲G̲C̲T̲A̲C̲T̲T̲T̲G̲C̲T̲A̲A̲-3′		
Ap-LC-51	5′-C̲T̲A̲T̲A̲G̲C̲A̲A̲T̲G̲G̲T̲A̲C̲G̲G̲T̲A̲C̲T̲T̲C̲C̲AAGCTGAGGGACACTGTCGCGCTCCAGGGACAT	3.5	109 ± 4.9
	ATCCGCGC̲A̲A̲A̲A̲G̲T̲G̲C̲A̲C̲G̲C̲T̲A̲C̲T̲T̲T̲G̲C̲T̲A̲A̲-3′		
Ap-LC-54	5′-C̲T̲A̲T̲A̲G̲C̲A̲A̲T̲G̲G̲T̲A̲C̲G̲G̲T̲A̲C̲T̲T̲C̲C̲AAGCTATCGCTCATCTGCACGTAAGCCCTCTGGT	1.5	125 ± 7.4
	TAGGTCC̲A̲A̲A̲A̲G̲T̲G̲C̲A̲C̲G̲C̲T̲A̲C̲T̲T̲T̲G̲C̲T̲A̲A̲-3′		
Ap-LC-69	5′-C̲T̲A̲T̲A̲G̲C̲A̲A̲T̲G̲G̲T̲A̲C̲G̲G̲T̲A̲C̲T̲T̲C̲C̲GGACGCGGTTAGCGCAGAATTTAACTACCAAAG	2	99 ± 4.6
	GGGTTTAC̲A̲A̲A̲A̲G̲T̲G̲C̲A̲C̲G̲C̲T̲A̲C̲T̲T̲T̲G̲C̲T̲A̲A̲-3′		
Ap-LC-76	5′-C̲T̲A̲T̲A̲G̲C̲A̲A̲T̲G̲G̲T̲A̲C̲G̲G̲T̲A̲C̲T̲T̲C̲C̲CTTTGTGCGGCCACGTGCCGTAGATTTGGGTTTA	1	89 ± 6.9
	AGCGCTC̲A̲A̲A̲A̲G̲T̲G̲C̲A̲C̲G̲C̲T̲A̲C̲T̲T̲T̲G̲C̲T̲A̲A̲-3′		

**Fig. 2:**
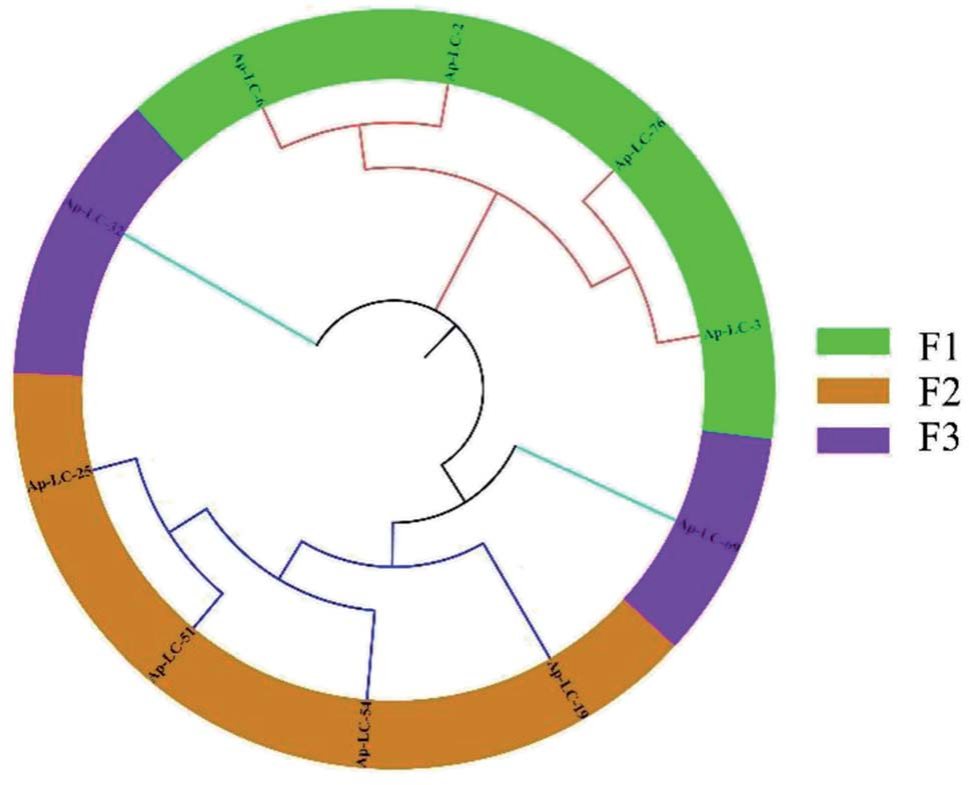
Phylogenetic tree, alignment and clustering. A DNAMAN phylogenetic tree analysis of the 88 bp DNA sequences from 10 diferent aptamer sequences.

The 88 bp random area sequences of the ten candidate aptamers underwent homologous alignment and multiple sequence alignment using DNAMAN software. The sequencing result was consistent with the length of the initial ssDNA library. The results showed that the sequence homology reached 66.98%. In addition, the obtained aptamers were rich in G and C sequences. Finally, among the 88 bp sequences identified, multiple sequence alignments and phylogenetic analysis revealed that three groups of sequences (F1, F2 and F3) were distributed (Fig. 2).

**Fig. 3:**
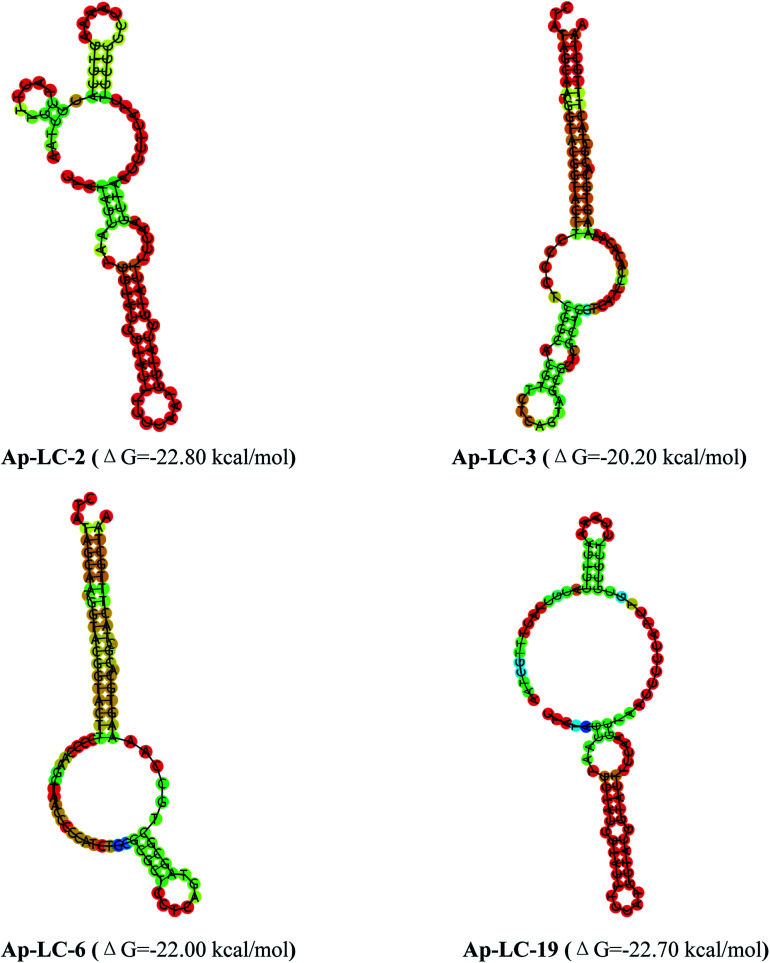
Structural analysis of the four aptamers were performed using mfold to predict the secondary structures.

The secondary structures of the candidate aptamers were predicted by using the NUPACK software, based on the principle of minimum folding energy. The predicted secondary structures of the candidate aptamers had abundant secondary structures, which were mainly based on the typical stable loop-stem and bulge structures. The results indicate that the stem-loop structures^30,31^ could play a major role in their binding to target serum. The predicted secondary structures of aptamers Ap-LC-2, Ap-LC-3, Ap-LC-6 and Ap-LC-19 with minimum free energies (Δ*G*) of −20.20 and −22.80 kcal mol^−1^, respectively, are shown in Fig. 3.

**Fig. 4:**
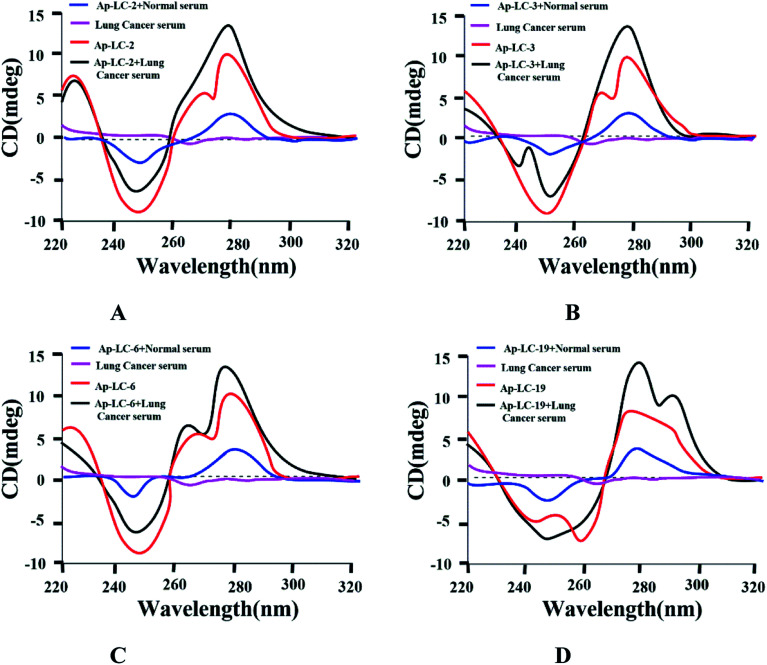
CD spectrum identification of binding activity between four aptamers and lung cancer serum.

To confirm the binding activity between lung cancer serum and aptamers, conformation changes of the candidate aptamer before and after lung cancer serum binding were identified by CD spectra. Fig. 4 shows that lung cancer serum has an extremely weak CD signal. The four aptamers displayed a positive and negative peak at about 275 nm and 250 nm, respectively, signifying a stem loop type B-DNA structure.^32^ When the aptamer-lung cancer serum complex was formed by the binding of lung cancer serum with the aptamer, the positive peak at 275 nm was significantly increased, while the negative peak at 250 nm was only slightly changed. When the aptamer-normal serum complex was formed by the binding of normal serum with the aptamer, the positive peak and the negative peak were only slightly changed. Therefore, the aptamer spatial conformation changed after binding with lung cancer serum, which confirmed the interaction between these aptamers and target serum, respectively.

### Assessment of aptamer binding to lung cancer serum

**Fig. 5:**
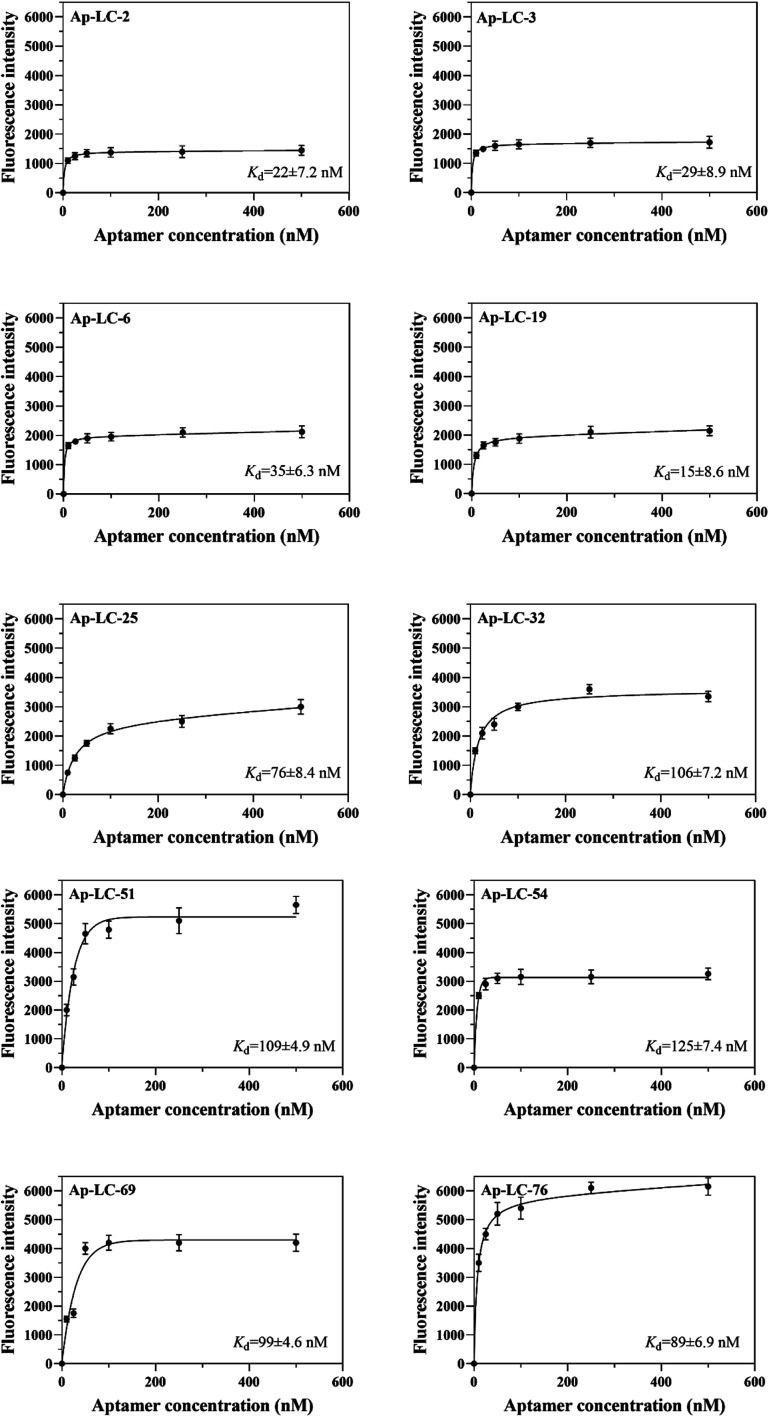
Binding saturation curve of the pooled lung cancer aptamers.

To choose sufficient binding sequences, the binding affinities of the chosen candidate aptamers were measured by fluorescence assay using the chemically synthesized 5-FAM labeled aptamers. The *K*_d_ value was calculated using Sigma plot 12.5, and we found that *K*_d_ values of Ap-LC-2, Ap-LC-3, Ap-LC-6, Ap-LC-19, Ap-LC-25, Ap-LC-32, Ap-LC-51, Ap-LC-54, Ap-LC-69 and Ap-LC-76 are respectively 22 ± 7.2 nM, 29 ± 8.9 nM, 35 ± 6.3 nM, 15 ± 8.6 nM, 76 ± 8.4 nM, 106 ± 7.2 nM, 109 ± 4.9 nM, 125 ± 7.4 nM, 99 ± 4.6 nM, 89 ± 6.9 nM, respectively (Fig. 5). The lower *K*_d_ value represented a higher affinity. Therefore, the results of the binding assay test and assessment of aptamer binding to pooled lung cancer serum experiment further confirmed that Ap-LC-19 could be used as the strongest capability on target binding to pooled lung cancer serum among these four aptamers. Additionally, Ap-LC-19 aptamer was validated to be the optimal aptamers among these four aptamers, which may be developed into new probes for tumor serum targeting and imaging and be used for cancer diagnosis and therapy.

### Specificity test

**Fig. 6:**
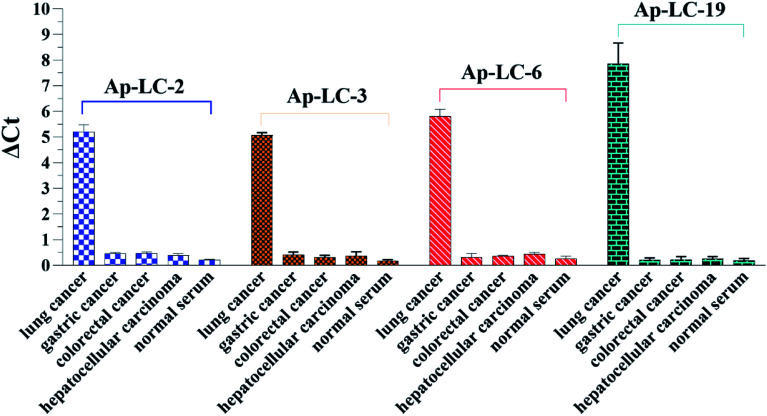
Specificity test of the aptamer-based assay using qPCR detection toward normal human serum sample, several different cancer serum samples gastric cancer, colorectal cancer, hepatocellular carcinoma (each of 200 μL).

The specificity of Ap-LC-2, Ap-LC-3, Ap-LC-6 and Ap-LC-19 to other tumor sera and normal serum were further measured and monitored using qPCR. We used Δ*C*_t_ value to quantify the signals, as it directly relates to the relative difference in the nucleic acid template copy number between two samples. We found that the Δ*C*_t_ values of the other tumor serum and normal serum were almost all less than 0.5. The Δ*C*_t_ value of those tumor sera is shown in Table S2.† In addition, a Δ*C*_t_ value of more than 5 was observed between the lung cancer serum and the other serum target. There were many more aptamers bound to the lung cancer serum-MBs than the other 4 types of serum-MBs, suggesting that these four aptamers could specifically bind to lung cancer serum. Among all the tested tumor serum and normal serum, Ap-LC-2, Ap-LC-3, Ap-LC-6 and Ap-LC-19 bound only to lung cancer serum, and did not bind to other serum (such as gastric cancer, colorectal cancer and hepatocellular carcinoma and normal serum). This demonstrates that our obtained aptamers had a good selectivity for the discrimination of lung cancer from other cancer serum (Fig. 6). These results indicate that Ap-LC-19 could be the suitable aptamer of lung cancer serum and could be used as a recognition element in aptamer-based biosensors for efficient early diagnosis of lung cancer or as a potential tool for targeted therapy.

### Using the selected aptamers as a capture probe in detecting clinical samples

**Fig. 7:**
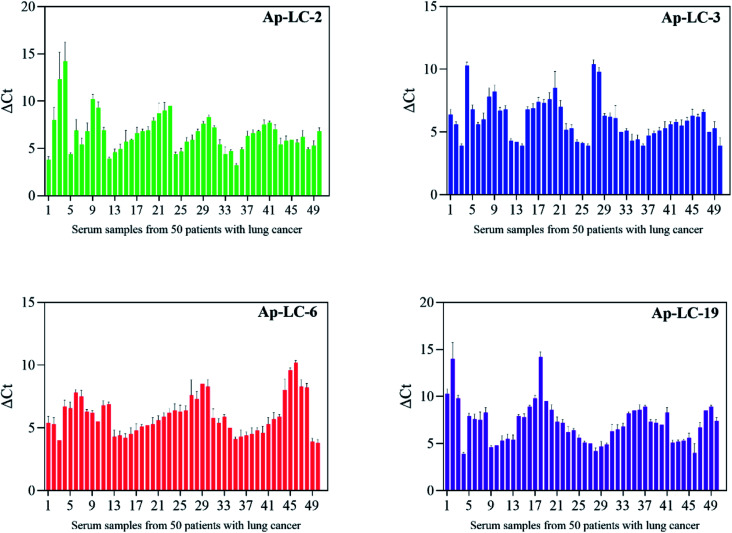
Sera were collected from 50 lung cancer patients and 50 healthy controls for clinical testing.

For the specificity analysis using the Ap-LC-2, Ap-LC-3, Ap-LC-6 and Ap-LC-19 as a detection probe, respectively, the four DNA aptamers were incubated with serum-MBs of fifty cases of lung cancer sera, and the amount of bound aptamer was analyzed by qPCR. A negative control normal serum was performed simultaneously. Fifty cases of normal sera were specifically identified according to the above steps. qPCR was used to monitor the binding of aptamer and serum process according to the Δ*C*_t_ values. As shown in Fig. 7, a Δ*C*_t_ value of 4–15 was observed between the lung cancer serum and the normal serum. The results showed that we successfully screened the aptamer of lung cancer serum with high specificity and strong affinity. The *C*_t_ value of healthy control serum is shown in Table S3.†

### Identification of candidate serological biomarker

**Fig. 8:**
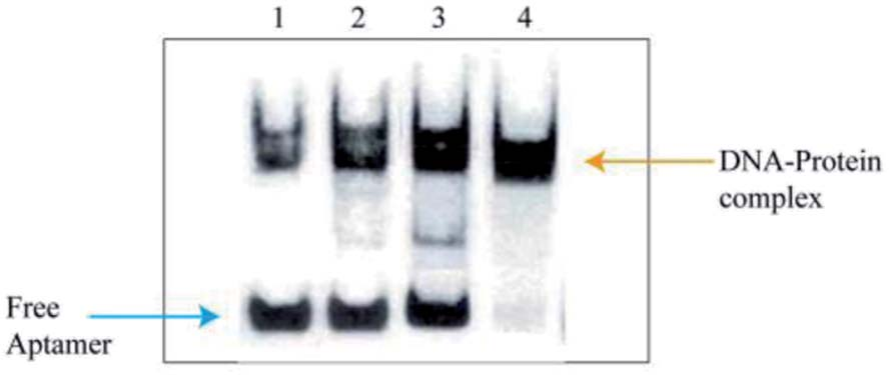
Electrophoretic mobility shift assay (EMSA) identification results. (1) the pooled normal serum and initial ssDNA library, (2) the pooled lung cancer serum and initial ssDNA library, (3) the pooled normal serum and Apt-LC-19, (4) the pooled lung cancer serum and Apt-LC-19.

The slowly migrated band enriched the Apt-LC-19 aptamer and the pooled lung cancer serum complexes in electrophoretic mobility shift assay (EMSA) were excised and the protein components in it were recovered and were subjected to MS analysis. The corresponding position in the control lane (the complex of the initial ssDNA library and pooled normal serum, the complex of the initial ssDNA library and pooled lung cancer serum, the complex of the Apt-LC-19 and pooled normal serum, the complex of the Apt-LC-19 and pooled lung cancer serum) was excised and identified by MS simultaneously to exclude the protein mobilized similarly with the complex. As shown in Fig. 8, the initial ssDNA library as a random sequence does not specifically bind to pooled lung cancer serum. However, the Apt-LC-19 aptamer specifically recognized lung cancer serum without binding to normal serum. We performed MALDI-TOF MS followed by secondary peptide sequencing MS analysis for the identification of the enriched protein. The CLEC3B match in the control band. CLEC3B (UniProt accession number sp|P02768; peptide: WTPYQGCEALCCPEPK) was detected from lung cancer serum. CLEC3B could be useful biomarkers for early detection of lung cancer and in monitoring its evolution. CLEC3B had already been reported as a useful biomarker for the diagnosis of lung cancer. It was shown to be up-regulated in lung cancer patients by ELISA.^33^

## Conclusions

Currently, novel therapeutic and diagnostic tools have been developed across the world. Early detection and treatment of lung cancer is crucial to improving the survival rate of patients with lung cancer. The serum contains a wealth of histological information related to early diagnosis of disease. The vitro detection of serum in clinical samples is of great importance. Whole serum are important targets of aptamers. Therefore, it is meaningful for us to obtain the specific reagent and to establish an effective method for lung cancer detection. Aptamers represent a new generation of molecular recognition elements with applicability to the selective capture and detection of target molecules for the development of highly sensitive, highly specific, and more rapid diagnostic reagents.

In the present study, we used a new modified SELEX (named serum-SELEX) to isolate aptamer that can specifically bind lung cancer serum, without any prior knowledge of their target. We designed a serum-SELEX strategy to generate a group of serum aptamers valuable for lung cancer diagnosis and with translational potential, which may facilitate the clinical diagnosis of lung cancer based on serum aptamers. All of them were verified to be specific to lung cancer serum to different extents. Four aptamers were further evaluated with an enlarged size of clinical serum specimens and proven to be valuable for lung cancer diagnosis. We found that Ap-LC-19 is the optimal aptamer probe with the lowest dissociation constant (*K*_d_) values of 15 ± 8.6 nM and higher specificity assessed by qPCR among the four candidates after performance comparison and verification.

Secondary structure analysis indicated that the minimum free energy was less than −22.80 kcal mol^−1^ for Ap-LC-2 and −22.70 kcal mol^−1^, −22.00 kcal mol^−1^ and −20.20 kcal mol^−1^ for Ap-LC-19, Ap-LC-6 and Ap-LC-3 which further support our findings on the higher binding affinity of Ap-LC-19 aptamer. The aptamers, Ap-LC-2, Ap-LC-3, Ap-LC-6 and Ap-LC-19 with definite stem–loop structures in the random region were examined to determine their affinity to the target serum.

Here, these aptamers show great potential for lung cancer in targeted diagnosis and treatment. We also performed MALDI-TOF MS followed by secondary peptide sequencing MS analysis for the identification of the aptamer targeted proteins. CLEC3B could be useful biomarkers for early detection of lung cancer and in monitoring its evolution. In addition, this study demonstrated the great potential of the aptamer of lung cancer serum for early lung cancer in targeted diagnosis and treatment, and provided an alternative select strategy for aptamer selection against complex molecules reagents, which can accelerate the exploration and characterization of the human serum proteome.

## Conflicts of interest

There are no conflicts to declare.

## Supplementary Material

RA-011-D1RA06233F-s001
