# Supplementary material for: Identification of a novel DNA aptamer that selectively targets lung cancer serum
Source: RSC Adv. 2021 Oct 15;11(53):33759–69. doi: 10.1039/d1ra06233f (PMC9042271; doi:10.1039/d1ra06233f)
Supplement: RA-011-D1RA06233F-s001 [file RA-011-D1RA06233F-s001.pdf]

Electronic Supplementary Material (ESI) for RSC Advances.
This journal is © The Royal Society of Chemistry 2021

Table S-1. List of serum samples used in this study.

ID	Clinical status
N1	Healthy control
N2	Healthy control
N3	Healthy control
N4	Healthy control
N5	Healthy control
N6	Healthy control
N7	Healthy control
N8	Healthy control
N9	Healthy control
N10	Healthy control
N11	Healthy control
N12	Healthy control
N13	Healthy control
N14	Healthy control
N15	Healthy control
N16	Healthy control
N17	Healthy control
N18	Healthy control
N19	Healthy control
N20	Healthy control
N21	Healthy control
N22	Healthy control
N23	Healthy control
N24	Healthy control
N25	Healthy control
N26	Healthy control
N27	Healthy control
N28	Healthy control
N29	Healthy control
N30	Healthy control
N31	Healthy control
N32	Healthy control
N33	Healthy control
N34	Healthy control
N35	Healthy control
N36	Healthy control
N37	Healthy control
N38	Healthy control
N39	Healthy control
N40	Healthy control
N41	Healthy control

N42	Healthy control
N43	Healthy control
N44	Healthy control
N45	Healthy control
N46	Healthy control
N47	Healthy control
N48	Healthy control
N49	Healthy control
N50	Healthy control
N51	Healthy control
N52	Healthy control
N53	Healthy control
N54	Healthy control
N55	Healthy control
N56	Healthy control
N57	Healthy control
N58	Healthy control
N59	Healthy control
N60	Healthy control
N61	Healthy control
N62	Healthy control
N63	Healthy control
N64	Healthy control
N65	Healthy control
N66	Healthy control
N67	Healthy control
N68	Healthy control
N69	Healthy control
N70	Healthy control
N71	Healthy control
N72	Healthy control
N73	Healthy control
N74	Healthy control
N75	Healthy control
N76	Healthy control
N77	Healthy control
N78	Healthy control
N79	Healthy control
N80	Healthy control
N81	Healthy control
N82	Healthy control
N83	Healthy control
N84	Healthy control
N85	Healthy control

N86	Healthy control
N87	Healthy control
N88	Healthy control
N89	Healthy control
N90	Healthy control
N91	Healthy control
N92	Healthy control
N93	Healthy control
N94	Healthy control
N95	Healthy control
N96	Healthy control
N97	Healthy control
N98	Healthy control
N99	Healthy control
N100	Healthy control
LC-1	Lung cancer
LC-2	Lung cancer
LC-3	Lung cancer
LC-4	Lung cancer
LC-5	Lung cancer
LC-6	Lung cancer
LC-7	Lung cancer
LC-8	Lung cancer
LC-9	Lung cancer
LC-10	Lung cancer
LC-11	Lung cancer
LC-12	Lung cancer
LC-13	Lung cancer
LC-14	Lung cancer
LC-15	Lung cancer
LC-16	Lung cancer
LC-17	Lung cancer
LC-18	Lung cancer
LC-19	Lung cancer
LC-20	Lung cancer
LC-21	Lung cancer
LC-22	Lung cancer
LC-23	Lung cancer
LC-24	Lung cancer
LC-25	Lung cancer
LC-26	Lung cancer
LC-27	Lung cancer
LC-28	Lung cancer
LC-29	Lung cancer

LC-30	Lung cancer
LC-31	Lung cancer
LC-32	Lung cancer
LC-33	Lung cancer
LC-34	Lung cancer
LC-35	Lung cancer
LC-36	Lung cancer
LC-37	Lung cancer
LC-38	Lung cancer
LC-39	Lung cancer
LC-40	Lung cancer
LC-41	Lung cancer
LC-42	Lung cancer
LC-43	Lung cancer
LC-44	Lung cancer
LC-45	Lung cancer
LC-46	Lung cancer
LC-47	Lung cancer
LC-48	Lung cancer
LC-49	Lung cancer
LC-50	Lung cancer
LC-51	Lung cancer
LC-52	Lung cancer
LC-53	Lung cancer
LC-54	Lung cancer
LC-55	Lung cancer
LC-56	Lung cancer
LC-57	Lung cancer
LC-58	Lung cancer
LC-59	Lung cancer
LC-60	Lung cancer
LC-61	Lung cancer
LC-62	Lung cancer
LC-63	Lung cancer
LC-64	Lung cancer
LC-65	Lung cancer
LC-66	Lung cancer
LC-67	Lung cancer
LC-68	Lung cancer
LC-69	Lung cancer
LC-70	Lung cancer
LC-71	Lung cancer
LC-72	Lung cancer
LC-73	Lung cancer

LC-74	Lung cancer
LC-75	Lung cancer
LC-76	Lung cancer
LC-77	Lung cancer
LC-78	Lung cancer
LC-79	Lung cancer
LC-80	Lung cancer
LC-81	Lung cancer
LC-82	Lung cancer
LC-83	Lung cancer
LC-84	Lung cancer
LC-85	Lung cancer
LC-86	Lung cancer
LC-87	Lung cancer
LC-88	Lung cancer
LC-89	Lung cancer
LC-90	Lung cancer
LC-91	Lung cancer
LC-92	Lung cancer
LC-93	Lung cancer
LC-94	Lung cancer
LC-95	Lung cancer
LC-96	Lung cancer
LC-97	Lung cancer
LC-98	Lung cancer
LC-99	Lung cancer
LC-100	Lung cancer
GC-1	Gastric cancer
GC-2	Gastric cancer
GC-3	Gastric cancer
GC-4	Gastric cancer
GC-5	Gastric cancer
GC-6	Gastric cancer
GC-7	Gastric cancer
GC-8	Gastric cancer
GC-9	Gastric cancer
GC-10	Gastric cancer
CC-1	Colorectal cancer
CC-2	Colorectal cancer
CC-3	Colorectal cancer
CC-4	Colorectal cancer
CC-5	Colorectal cancer
CC-6	Colorectal cancer
CC-7	Colorectal cancer

CC-8	Colorectal cancer
CC-9	Colorectal cancer
CC-10	Colorectal cancer
HCC-1	Hepatocellular carcinoma
HCC-2	Hepatocellular carcinoma
HCC-3	Hepatocellular carcinoma
HCC-4	Hepatocellular carcinoma
HCC-5	Hepatocellular carcinoma
HCC-6	Hepatocellular carcinoma
HCC-7	Hepatocellular carcinoma
HCC-8	Hepatocellular carcinoma
HCC-9	Hepatocellular carcinoma
HCC-10	Hepatocellular carcinoma

Table S-2. Specicity test of the **Δ**Ct value of those tumor sera

ID	Clinical status	ΔCt value
GC-1	Gastric cancer	0.25
GC-2	Gastric cancer	0.35
GC-3	Gastric cancer	0.5
GC-4	Gastric cancer	0.45
GC-5	Gastric cancer	0.36
GC-6	Gastric cancer	0.14
GC-7	Gastric cancer	0.39
GC-8	Gastric cancer	0.36
GC-9	Gastric cancer	0.12
GC-10	Gastric cancer	0.11
CC-1	Colorectal cancer	0.25
CC-2	Colorectal cancer	0.45
CC-3	Colorectal cancer	0.39
CC-4	Colorectal cancer	0.1
CC-5	Colorectal cancer	0.15
CC-6	Colorectal cancer	0.12
CC-7	Colorectal cancer	0.19
CC-8	Colorectal cancer	0.39
CC-9	Colorectal cancer	0.47
CC-10	Colorectal cancer	0.26
HCC-1	Hepatocellular carcinoma	0.49
HCC-2	Hepatocellular carcinoma	0.5
HCC-3	Hepatocellular carcinoma	0.26
HCC-4	Hepatocellular carcinoma	0.23
HCC-5	Hepatocellular carcinoma	0.27
HCC-6	Hepatocellular carcinoma	0.35

HCC-7	Hepatocellular carcinoma	0.39
HCC-8	Hepatocellular carcinoma	0.11
HCC-9	Hepatocellular carcinoma	0.16
HCC-10	Hepatocellular carcinoma	0.1

Table S-3. Specicity test of the Ct value of healthy control serum

Healthy control	Ct value
N51	39.6
N52	38.7
N53	40
N54	39.2
N55	38.6
N56	40
N57	40
N58	39.9
N59	39
N60	40
N61	40
N62	40
N63	39.8
N64	40
N65	40
N66	40
N67	39
N68	39.4
N69	39.7
N70	40
N71	40
N72	40
N73	40
N74	39.5
N75	39.7
N76	40
N77	40
N78	40
N79	39.9
N80	39.5
N81	39.1
N82	40
N83	40
N84	39.5
N85	40
N86	40

N87	40
N88	40
N89	40
N90	39.7
N91	39.6
N92	39.1
N93	40
N94	40
N95	40
N96	40
N97	40
N98	40
N99	40
N100	40
